# Intermediate scattering functions of a rigid body monoclonal antibody protein in solution studied by dissipative particle dynamic simulation

**DOI:** 10.1063/4.0000086

**Published:** 2021-04-08

**Authors:** Yanqin Zhai, Nicos S. Martys, William L. George, Joseph E. Curtis, Jannatun Nayem, Y Z, Yun Liu

**Affiliations:** 1 Department of Nuclear, Plasma, and Radiological Engineering, University of Illinois at Urbana-Champaign, 104 South Wright Street, Urbana, Illinois 61801, USA; 2 Beckman Institute for Advanced Science and Technology, University of Illinois at Urbana-Champaign, 405 North Mathew Avenue, Urbana, Illinois 61801, USA; 3 Center for Biophysics and Quantitative Biology, University of Illinois at Urbana-Champaign, 600 South Mathews Avenue, Urbana, Illinois 61801, USA; 4 Program of Computational Science and Engineering, 1205 West Clark Street, University of Illinois at Urbana-Champaign, Urbana, Illinois 61801, USA; 5 Materials and Construction Research Division of Engineering Laboratory, National Institute of Standards and Technology, 100 Bureau Drive, Gaithersburg, Maryland 20899, USA; 6 Information Technology Laboratory, National Institute of Standards and Technology, 100 Bureau Drive, Gaithersburg, Maryland 20899, USA; 7 NIST Center for Neutron Research, National Institute of Standards and Technology, 100 Bureau Drive, Gaithersburg, Maryland 20899, USA; 8 Department of Chemical and Biomolecular Engineering, University of Delaware, 150 Academy Street, Newark, Delaware 19716, USA; 9 Department of Electrical and Computer Engineering, University of Illinois at Urbana-Champaign, 306 North Wright Street, Urbana, Illinois 61801, USA; 10 Department of Physics and Astronomy, University of Delaware, 217 Sharp Lab, Newark, Delaware 19716, USA

## Abstract

In the past decade, there was increased research interest in studying internal motions of flexible proteins in solution using Neutron Spin Echo (NSE) as NSE can simultaneously probe the dynamics at the length and time scales comparable to protein domain motions. However, the collective intermediate scattering function (ISF) measured by NSE has the contributions from translational, rotational, and internal motions, which are rather complicated to be separated. Widely used NSE theories to interpret experimental data usually assume that the translational and rotational motions of a rigid particle are decoupled and independent to each other. To evaluate the accuracy of this approximation for monoclonal antibody (mAb) proteins in solution, dissipative particle dynamic computer simulation is used here to simulate a rigid-body mAb for up to about 200 ns. The total ISF together with the ISFs due to only the translational and rotational motions as well as their corresponding effective diffusion coefficients is calculated. The aforementioned approximation introduces appreciable errors to the calculated effective diffusion coefficients and the ISFs. For the effective diffusion coefficient, the error introduced by this approximation can be as large as about 10% even though the overall agreement is considered reasonable. Thus, we need to be cautious when interpreting the data with a small signal change. In addition, the accuracy of the calculated ISFs due to the finite computer simulation time is also discussed.

## I. INTRODUCTION

The Neutron Spin Echo (NSE) Spectroscopy was first invented by Ferenc Mezei in the 1970s.^1^ Different from other inelastic neutron scattering techniques, NSE directly measures the intermediate scattering function (ISF) as a function of momentum transfer and correlation time. By taking advantage of the spin procession of a neutron in a magnet field, it can probe the time scale from several picoseconds to hundreds of nanoseconds with the length scale from a few angstroms to tens of nanometers. Owing to the large time and length scales it can probe, it has been used in many scientific areas including material sciences,^2,3^ liquid physics,^4–6^ glass transitions,^7,8^ polymer dynamics,^9–11^ and biological systems,^12^ especially protein clusters^13–16^ and protein internal motions.^17–22^ Due to the interest in understanding the relationship between protein motions and its functionalities, protein internal motions are intensively studied by NSE in the past decade.^23,24^

For macromolecules in solution, the coherent intermediate scattering function, *I*(*Q*, *t*), measured by NSE contains the information of translational, rotational, and internal domain motions.^17,25,26^ Here, *Q* is the magnitude of the wavevector transfer and *t* is the correlation time.^25^ It is highly non-trivial to separate different types of motions from *I*(*Q*, *t*). Different models have been proposed.^17,18,22,25,26^ One commonly used approach is to assume that the translation, rotational, and internal domain motions are independent of each other so that *I*(*Q*, *t*) is simply the multiplication of the intermediate scattering functions from each different type of motions.^22,24,26^ However, the accuracy of this approximation has not been rigorously evaluated for anisotropic particles.^22^

One consequence of this assumption is that the total diffusion coefficient, $D_{\text{eff}}(Q)$, obtained from *I*(*Q*, *t*) is simply the summed values of two contributions: one is the diffusion due to a rigid body model of the protein due to the translational and rotational motions and another one is due to the internal domain motion.^17,18,22,25^ The accuracy of the obtained internal dynamics thus depends on the accuracy of the estimation of $D_{\text{eff}}(Q)$ based on a rigid protein model.^22^ However, the contribution of internal protein motions to the total effective diffusion coefficient is usually very small (about ≈10% of $D_{\text{eff}}(Q)$).^17,18^ This makes it very important to investigate the accuracy of the estimated $D_{\text{eff}}(Q)$ of a rigid body protein model based on the aforementioned approximation.

While there are different ways to separate the translational and rotational motions for rigid proteins, one widely used approach is to assume that the center-of-mass and rotational motions are independent from each other.^17,18,24–26^ Here, we evaluate the accuracy of this approximation by studying a model monoclonal antibody (mAb) provided by the National Institute of Standards and Technology (NISTmAb), which is a reference material introduced to support the pharmaceutical industry.^27^ The mAb based therapeutic drugs have been the fastest growing sector of the pharmaceutical industry in the past decade. Also, their global sales reached 105 billion dollars in 2016.^28,29^ NSE has been used to study the cluster formation and internal motions of different mAbs.^14,16,20^ Due to the similarity of the overall shape of mAbs, the understanding of NISTmAb can also be useful to study many other therapeutic mAbs.

In this work, we treat the NISTmAb as a rigid protein by constructing a coarse grained rigid model protein from its PDB structure. We performed Brownian motion simulations on this rigid-body protein model using the dissipative particle dynamics (DPD) simulation to evaluate the accuracy of the aforementioned approximation to model *I*(*Q*, *t*). The simulation was done on a single protein so that there is no need to worry about inter-protein motions for concentrated protein solutions.^25^ The contributions to *I*(*Q*, *t*) by the center-of-mass and rotational motions are evaluated. The effective diffusion coefficients as a function of *Q* are obtained by fitting *I*(*Q*, *t*). Our results indicate that at the short time limit, the rotational and center-of-mass motions are still coupled. Treating them as independent motions can result in up to about 10% deviation of the obtained $D_{\text{eff}}(Q)$. The comparison between the total collective ISF and the product of the center-of-mass and rotational ISFs shows quantitative difference. However, given the error bars of typical NSE experiments, the agreement seems to be acceptable even though one has to be careful when interpreting the small difference from the data.

## II. THEORIES

NSE typically measures the coherent ISF expressed as

$$\begin{array}{c} I(\vec{Q},t)=\frac{1}{V}\sum_{m,n}b_{m}b_{n}e^{i\vec{Q}\cdot \left[{\vec{r}}_{m}(0)-{\vec{r}}_{n}(t)\right]}\\ =\frac{1}{V}\left(\sum_{m}b_{m}e^{i\vec{Q}\cdot {\vec{r}}_{m}(0)}\right){\left(\sum_{n}b_{n}e^{i\vec{Q}\cdot {\vec{r}}_{n}(t)}\right)}^{*} \end{array},$$ (1)

where $\vec{Q}$ is the wave vector transfer, *V* is the volume of the system exposed to the neutron beam, *b_m_* and *b_n_* are the scattering lengths of the *m*th and the *n*th atoms, respectively, and ${\vec{r}}_{m}(0)$ and ${\vec{r}}_{n}(t)$ are the position of the *m*th atom at time *t* = 0 and the position of the *n*th atom at time *t*.

For macromolecules, such as proteins in solution, we can rewrite the term $\sum_{n}b_{n}e^{i\vec{Q}\cdot {\vec{r}}_{n}(t)}$ in Eq. (1) as $\sum_{k}F_{k}(\vec{Q},t)e^{i\vec{Q}\cdot {\vec{r}}_{k}(t)}$, where ${\vec{r}}_{k}(t)$ is the center of mass coordinates of the *j*th protein at time *t*, and $F_{k}(\vec{Q},t)=\sum_{n}b_{n}e^{i\vec{Q}\cdot \left[{\vec{r}}_{n}(t)-{\vec{r}}_{j}(t)\right]}$ is the Fourier transformation of the density function of the *j*th protein. Therefore, Eq. (1) can be expressed as

$$\begin{array}{c} I(\vec{Q},t)=\frac{1}{V}\left[\sum_{j}F_{j}(\vec{Q},0)e^{i\vec{Q}\cdot {\vec{r}}_{j}(0)}\right]{\left[\sum_{k}F_{k}(\vec{Q},t)e^{i\vec{Q}\cdot {\vec{r}}_{k}(t)}\right]}^{*} \end{array}.$$ (2)

If there is only one protein, we can simplify Eq. (2) as

$$I(\vec{Q},t)=\frac{1}{V}F(\vec{Q},0)F^{*}(\vec{Q},t)e^{i\vec{Q}\cdot \left[{\vec{r}}_{c}(0)-{\vec{r}}_{c}(t)\right]},$$ (3)

where ${\vec{r}}_{c}(t)$ is the center of mass coordinates of the protein at time *t*.

For a real system at dilute concentrations, the interaction between proteins is negligible and we are able to focus on the ISF of individual proteins.^25^ Since proteins are typically randomly orientated in solution, we can take the angular average of Eq. (3) as^25^

$$\begin{array}{c} I(Q,t)={\left\langle I(\vec{Q},t)\right\rangle }_{\theta }=\frac{1}{V}{\left\langle F(\vec{Q},0)F^{*}(\vec{Q},t)e^{i\vec{Q}\cdot \Delta {\vec{r}}_{c}(t)}\right\rangle }_{\theta } \end{array},$$ (4)

where ${\left\langle \cdots \right\rangle }_{\theta }$ represents the angular average and $\Delta {\vec{r}}_{c}(t)={\vec{r}}_{c}(0)-{\vec{r}}_{c}(t)$ describes the center-of-mass motion of the protein.

In NSE experiments, the measured results are usually normalized to be^25^

$$\begin{array}{c} \frac{I(Q,t)}{I(Q,0)}=\frac{{\left\langle F(\vec{Q},0)F^{*}(\vec{Q},t)e^{i\vec{Q}\cdot \Delta {\vec{r}}_{c}(t)}\right\rangle }_{\theta }}{{\left\langle F(\vec{Q},0)F^{*}(\vec{Q},0)\right\rangle }_{\theta }}\\ ={\left\langle \frac{F(\vec{Q},0)F^{*}(\vec{Q},t)}{P(Q)}\cdot e^{i\vec{Q}\cdot \Delta {\vec{r}}_{c}(t)}\right\rangle }_{\theta } \end{array},$$ (5)

where ${\left\langle F(Q,0)F^{*}(Q,0)\right\rangle }_{\theta }$ is the form factor, *P*(*Q*).

As a general form, Eq. (5) contains all the protein motions: translational, rotational, and internal motions. Also, we can define

$$I_{\text{intra}}(\vec{Q},t)=\frac{F(\vec{Q},0)F^{*}(\vec{Q},t)}{P(Q)},$$ (6)

which contains the information of both the rotational and internal motions, and

$$I_{\text{com}}(\vec{Q},t)=e^{i\vec{Q}\cdot \Delta {\vec{r}}_{c}(t)},$$ (7)

which contains only the information of the center-of-mass motion. In this way, Eq. (5) can be represented as

$$\frac{I(Q,t)}{I(Q,0)}={\left\langle I_{\text{com}}(\vec{Q},t)I_{\text{intra}}(\vec{Q},t)\right\rangle }_{\theta }.$$ (8)

Notice that the center-of-mass motions are usually coupled with the rotational and internal motions in Eq. (8); it is complicated to separate the different contributions.

While there are different ways to simplify Eq. (8), one widely used approach is^18–20,23,26,30^

$$\frac{I(Q,t)}{I(Q,0)}\approx I_{\text{com}}(Q,t)I_{\text{intra}}(Q,t),$$ (9)

where

$$\begin{array}{c} I_{\text{com}}(Q,t)={\left\langle I_{\text{com}}(\vec{Q},t)\right\rangle }_{\theta }\\ ={\left\langle e^{i\vec{Q}\cdot \Delta {\vec{r}}_{c}(t)}\right\rangle }_{\theta }\\ =e^{-D_{0}Q^{2}t} \end{array}$$ (10)

and

$$\begin{array}{c} I_{\text{intra}}(Q,t)={\left\langle I_{\text{intra}}(\vec{Q},t)\right\rangle }_{\theta }\\ =\frac{{\left\langle F(\vec{Q},0)F^{*}(\vec{Q},t)\right\rangle }_{\theta }}{P(Q)}, \end{array}$$ (11)

where $I_{\text{com}}(Q,t)$ is for the center-of-mass motion, *D*_0_ is the free diffusion coefficient, and $I_{\text{intra}}(Q,t)$ is for both rotational and internal motions, respectively. Since the studied protein here is rigid, $I_{\text{intra}}(Q,t)$ is purely due to the rotational motion. Hence, in this paper, $I_{\text{intra}}(Q,t)$ = $I_{\text{rot}}(Q,t)$.

## III. DISSIPATIVE PARTICLE DYNAMICS SIMULATION

**FIG. 1.**
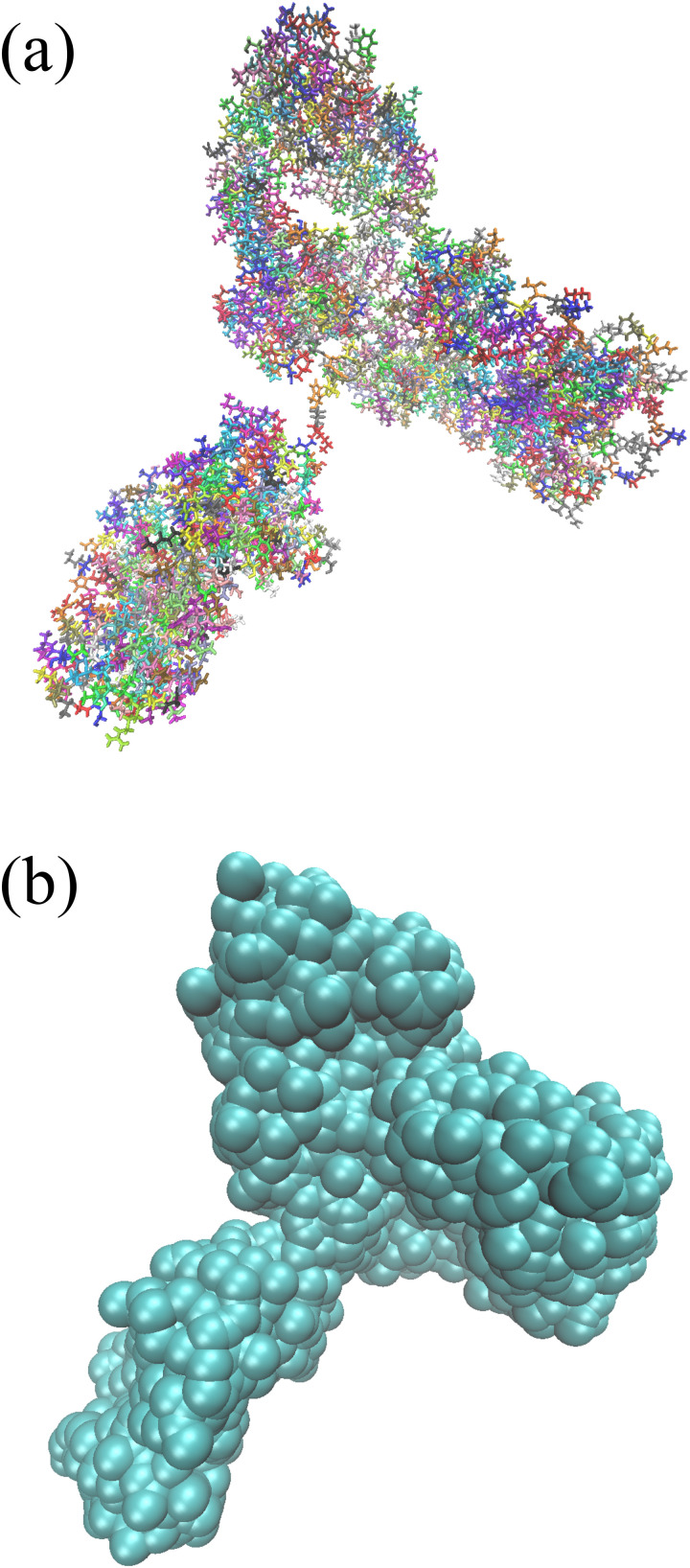
(a) 3D picture of the NISTmAb based on its PDB structure and (b) the rigid-body model used in the Brownian motion simulations. Each cyan sphere in the rigid-body model represents an amino acid residue.

Typically, mAb proteins are considered to be asymmetric Y-shaped particles. Our all-atom model of the NISTmAb was generated from coordinates derived from x-ray crystallography of the Fc and Fab domains with interdomain orientation from a representative single structure consistent with small-angle scattering data^29^ as shown in Fig. 1(a). A rigid-body model was generated using residue-based coarse graining using the Martini force field^31^ as shown in Fig. 1(b).

We use a dissipative particle dynamics (DPD)^32^ based approach for modeling the fluid phase. The simulation box size is approximately 6 times of the protein diameter. The simulation has been run long enough, which is estimated to correspond to about 200 ns for a mAb in water for each independent run. Compared with the molecular dynamics (MD) simulations, the DPD method introduces the random force and the hydrodynamic effect due to the motions of solvent molecules that affect the rotational and translational motions of a macromolecule in solution. Even though all atomic simulation including explicit water molecules can also include the hydrodynamic effect, the DPD method is simpler so that it can potentially simulate longer simulation time if needed.

DPD is a mesoscale model of fluids that can loosely be thought of as a Lagrangian formulation of Navier–Stokes equations with the inclusion of thermal fluctuations for modeling Brownian motions. The details of this computational model are beyond the scope of the paper.^33,34^ Here, we highlight its main features and focus on the relevant aspects of the models to this work. The DPD simulation is similar in structure to molecular dynamics simulations. However, instead of modeling all the molecular properties of the system, the motions of mesoscopic DPD particles that represent a coarse grained fluid are considered. The DPD particles are subject to three forces: conservative, dissipative, and random, with the total force, ${\vec{F}}_{ij}^{T}$, on a particle to be expressed as

$${\vec{F}}_{ij}^{T}={\vec{F}}_{ij}^{C}+{\vec{F}}_{ij}^{D}+{\vec{F}}_{ij}^{R}.$$ (12)

The conservative force, ${\vec{F}}_{ij}^{C}$, is a soft repulsive radial force, which decreases linearly with the center-to-center distance, $\left|{\vec{r}}_{i}-{\vec{r}}_{j}\right|$, between two DPD particles *i* and *j* whose amplitude is chosen so that the compressibility of the DPD fluid is close to that of water.^35^ The dissipative force, ${\vec{F}}_{ij}^{D}$, is proportional to the difference of velocities between DPD particles *i* and *j*, ${\vec{v}}_{i}-{\vec{v}}_{j}$, and acts to slow down their relative motion and produce a viscous effect. Here, *v* is the velocity of a particle. Finally, a random force, ${\vec{F}}_{ij}^{R}$ is added to control the temperature of the system and is the mechanism for Brownian motions via the thermal fluctuations. The dissipative and random forces control the viscosity of the fluid and maintain a well-defined temperature, and they are related by the fluctuation-dissipation theorem. For convenience, the energy scale is chosen such that *k_B_*T = 1. In addition parameters are chosen such that the fluid system possesses a Gibbs-Boltzmann equilibrium state in order that detailed balance is respected.^36^ This approach has been show to recover the Einstein intrinsic viscosity for hard spheres and has been validated for a variety of flow scenarios, including Pouiselle flow and Jeffery's orbits for sheared ellipsoids.^33^

## IV. RESULTS AND DISCUSSION

To understand the individual contributions of the center-of-mass and rotational motions to the total collective ISF, we separate the trajectory into the center-of-mass and the rotational part as

$$\begin{array}{c} {\vec{r}}_{\text{com}}(t)={\vec{r}}_{c}(t)\\ {\vec{r}}_{i,rot}(t)={\vec{r}}_{i}(t)-{\vec{r}}_{c}(t), \end{array}$$ (13)

where ${\vec{r}}_{i}(t),\;{\vec{r}}_{\text{com}}(t)$, and ${\vec{r}}_{i,rot}(t)$ describe the trajectories of overall motion, center-of-mass motion, and rotational motion, respectively. ${\vec{r}}_{c}(t)=\frac{1}{n}\sum_{i}{\vec{r}}_{i}(t)$ is the center-of-mass of the protein at time *t*. Here, the mass is assumed to be evenly distributed among amino acid residues.

Considering a large number of proteins existing in the real solution, we take an angular average on Eq. (1) as

$$\begin{array}{c} I(Q,t)={\left\langle I(\vec{Q},t)\right\rangle }_{\theta }\\ =\frac{1}{V}\sum_{m,n}b_{m}b_{n}\frac{\;\text{sin}(Qr_{m,n}(t))}{Qr_{m,n}(t)} \end{array},$$ (14)

where $r_{m,n}(t)=|{\vec{r}}_{m}(0)-{\vec{r}}_{n}(t)|$. The normalized total, center-of-mass, and rotational collective ISFs following Eq. (14) are computed with $b_{m}=b_{n}=1$ for the computational convenience.

**FIG. 2.**
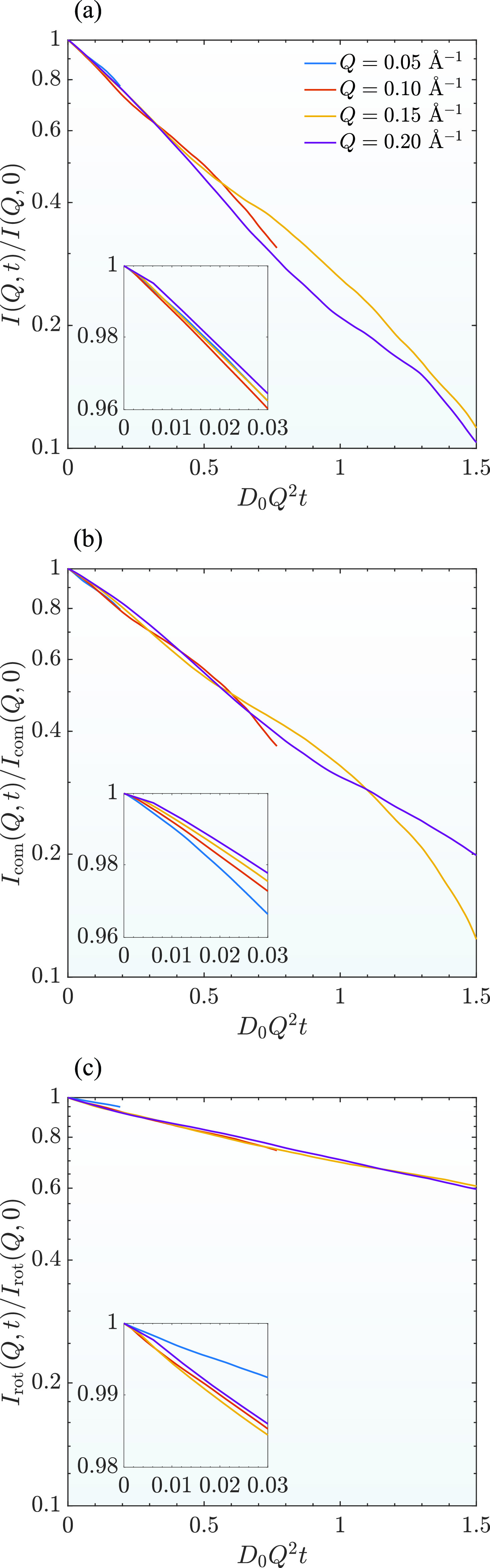
Normalized (a) total, (b) center-of-mass, and (c) rotational collective ISFs of the rigid-body protein calculated from the simulation trajectory at *Q* = 0.05 Å^−1^, 0.1 Å^−1^, 0.15 Å^−1^, and 0.2 Å^−1^.

The results are shown in Fig. 2. $I(Q,t)/I(Q,0),\;I_{\text{com}}(Q,t)/I_{\text{com}}(Q,0)$, and $I_{\text{rot}}(Q,t)/I_{\text{rot}}(Q,0)$ are the normalized ISFs for the total, center-of-mass, and rotational motions based on Eqs. (8), (10), and (11), respectively. The x-axis is plotted as a dimensionless value, $D_{0}Q^{2}t$, so that the results can be compared with those of other systems with different solvent viscosities. Results of four different *Q* values are shown in Fig. 2 with the value of $D_{0}Q^{2}t$ up to about 1.5. Here, $D_{0}$ is the free diffusion coefficient. The free diffusion coefficients of a mAb protein depend on the solvent viscosity with the value of about 3.7 Å^2^/ ns for a D_2_O based buffer. During NSE experiments for mAb samples, the interested *Q* range is from about 0.05 Å^−1^ to about 0.2 Å^−1^. The measured correlation time by a NSE for mAb systems ranges from about 1 ns to about 200 ns.^16^ The value of $D_{0}Q^{2}t$ for a NSE experiment may range from about 0.01 to about 2 at *Q* = 0.05 Å^−1^ while the value may range from about 0.2 to about 30 at *Q* = 0.2 Å^−1^.

$I(Q,t)/I(Q,0),\;I_{\text{com}}(Q,t)/I_{\text{com}}(Q,0)$, and $I_{\text{rot}}(Q,t)/I_{\text{rot}}(Q,0)$ are expected to be different at different *Q* values. For example, for a rigid spherical particle, $I(Q,t)/I(Q,0)=I_{\text{com}}(Q,t)/I_{\text{com}}(Q,0)=e^{-D_{0}Q^{2}t}$ for dilute protein solutions. Also, $I_{\text{rot}}(Q,t)/I_{\text{rot}}(Q,0)=1$ as NSE measures the coherent ISF. Note that this is different from the neutron scattering experiments focusing on incoherent ISF, for which $I_{\text{rot}}(Q,t)/I_{\text{rot}}(Q,0)$ decays and can be expressed as the summation of a series of exponential functions.^37^ Hence, if the coherent ISFs are plotted as a function of $D_{0}Q^{2}t$, all ISFs should be collapsed into a master curve for a rigid spherical particle. The difference between ISFs as a function of $D_{0}Q^{2}t$ is thus a direct reflection of the effect due to the anisotropic shape of a mAb protein.

**FIG. 3.**
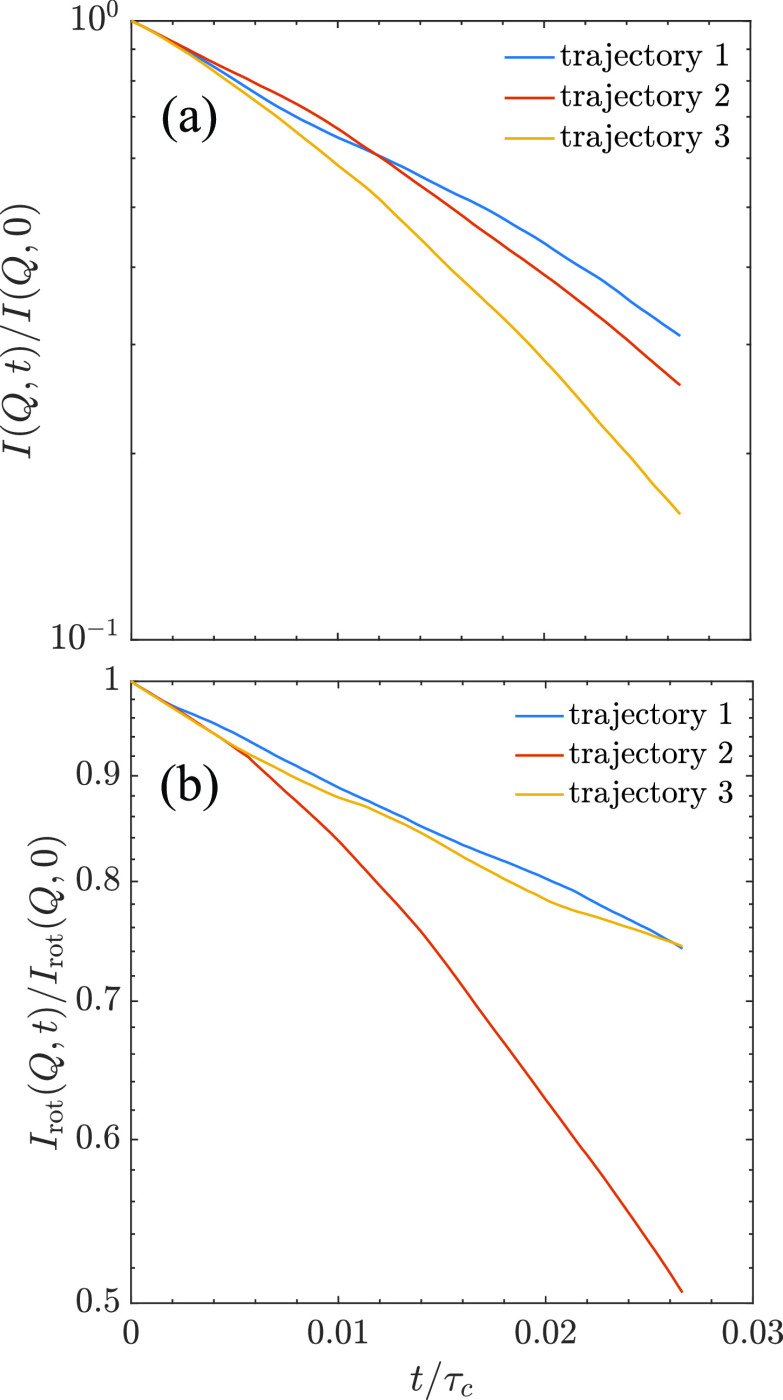
(a) Total and (b) rotational ISFs for three different trajectories of the NISTmAb at *Q* = 0.1 Å^−1^.

However, the ISFs in Fig. 2 do not show a consistent trend at relative large values of $D_{0}Q^{2}t$. This is most likely due to the fact that the simulation time is not long enough to sample sufficient Brownian motions. To verify this, three independent simulations with different initial values were conducted. Also, the ISFs from the three different sets of trajectories are calculated and shown in Fig. 3 as a function of $t/\tau_{c}$. Here, $\tau_{c}=\frac{\sigma }{6D_{0}}$ is the characteristic diffusion time and *σ* is the effective diameter of a particle. Thus, *τ_c_* is the time that needs a particle to diffuse a distance of its own diameter. Assuming σ= 10 nm and $D_{0}=3.7$ Å^2^/ns, *τ_c_* is about 400 ns for a mAb protein.

Theoretically speaking, as the three simulations are performed for the same system, the results should be similar to each other if each simulation time is long enough with sufficient sampling of the dynamics of the protein. However, for $t/\tau_{c}$ larger than about 0.003, the results in Fig. 3 start deviating from each other at the same *Q* value. As the Brownian motion is a stochastic process, it is expected that different simulations have different sampling of the particle motions. If the simulation time is long enough, the sampled motions resemble the true distribution function, and the results should be similar for the trajectories from different simulations. However, if the simulation time is too short, the sampled motions are biased by the particular simulation and may have different results as demonstrated here. Our simulation time for the three different trajectories is about 200 ns that corresponds to $t/\tau_{c}\approx 0.5$. Our results indicate that for $t/\tau_{c}$ less than about 0.003, the simulation results are similar to each other. The results obtained within this time region are reproducible with small uncertainty. For the calculated ISFs with $t/\tau_{c}>0.003$, the uncertainty due to the insufficient sampling makes it hard to compare the results from different trajectories. Note that for all atomic simulation with a protein, the typical simulation time is up to a few hundreds of nanoseconds,^38^ which is comparable to the simulation time used here. However, for this range of simulation time, it is not sufficient enough to extract *I*(*Q*, *t*) reliably for $t/\tau_{c}$ larger than about 0.003. Because of this, the difference of *I*(*Q*, *t*) at larger values of $D_{0}Q^{2}t$ shown in Fig. 2 is also partly due to the insufficient sampling of the simulation.

The effective diffusion coefficient, $D_{\text{eff}}(Q)$, has been widely used to understand the internal motions of proteins. $D_{\text{eff}}(Q)$ can be extracted from the collective ISF at the short-time limit through^15,17,25,26^

$$D_{\text{eff}}(Q)=-\frac{1}{Q^{2}}\underset{t\to 0}{\lim}\frac{\partial }{\partial t}\text{ln}\;[\frac{I(Q,t)}{I(Q,0)}].$$ (15)

**FIG. 4.**
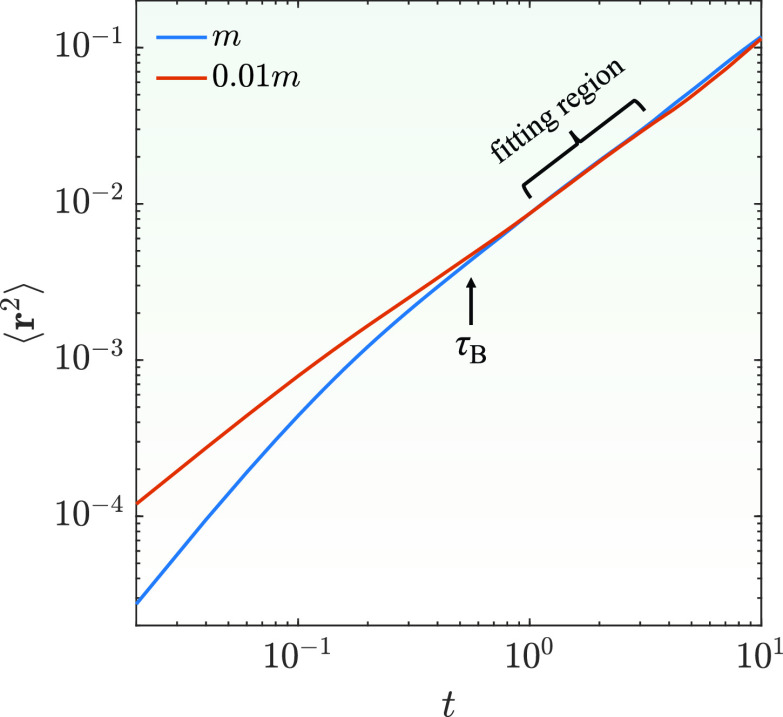
Center-of-mass mean square displacement of the rigid-body protein with the original mass and the mass scaled by 0.01. $\tau_{B}$ indicates the momentum relaxation time of the blue curve obtained from Eq. (16). Both *t* and $\left\langle r^{2}\right\rangle$ have dimensionless units used in the simulation.

Note that the correlation time for a short-time limit for a colloidal system should be larger than the momentum relaxation time so that the diffusion of a particle is independent of the mass and is only determined by its shape. To estimate the momentum relaxation time, *τ_B_*, the center-of-mass mean square displacement of the protein is calculated and shown as the blue line in Fig. 4. By fitting the curve to the analytical form of mean square displacement^39^

$$\left\langle r^{2}(t)\rangle =\tau_{B}^{2}{\left(1-e^{-t/\tau_{B}}\right)}^{2}(\nu_{0}^{2}-\frac{g}{2m\gamma })+\frac{g}{\gamma^{2}}[t-\tau_{B}(1-e^{-t/\tau_{B})}\right],$$ (16)

where $g=2\gamma k_{B}T$, *k_B_* is the Boltzmann constant, *T* is the temperature, *γ* is the friction coefficient, and *ν*_0_ is the initial velocity, we can extract $\tau_{B}=0.61$ in the unit of computer simulation time while *τ_c_* = 1880 in the unit of the computer simulation time. Hence, the relative time ratio for $\tau_{B}/\tau_{c}$ is about 0.0003. As a verification, we perform the same simulation with the mass of the protein changed by a factor of 0.01 and plot the result as the red line in Fig. 4. As we can observe, two lines superimpose at $t>\tau_{B}$ indicating that the diffusion of the protein is independent of the mass for *t* larger than the momentum relaxation time. Therefore, the center-of-mass motions of the protein have already entered the diffusive motion region after *τ_B_*. Later in the paper, the effective diffusion coefficients are obtained by fitting the ISF using Eq. (15) within the region where $t>\tau_{B}$, respectively. (Note that the fitting of the corresponding ISF is performed within the time region of t=1∼3 which is also well within the time region that the calculated ISFs are similar to each other based on the results from different simulation trajectories.) It is also noted that the decay of the intermediate scattering functions within such a short time is very small. However, with the accurate trajectories generated by the computer simulations, reliable results can be obtained. In a real experiment, the fitting time region for actual intermediate scattering functions has no such constraint as the experimental results are the ensemble of many proteins in solution.

**FIG. 5.**
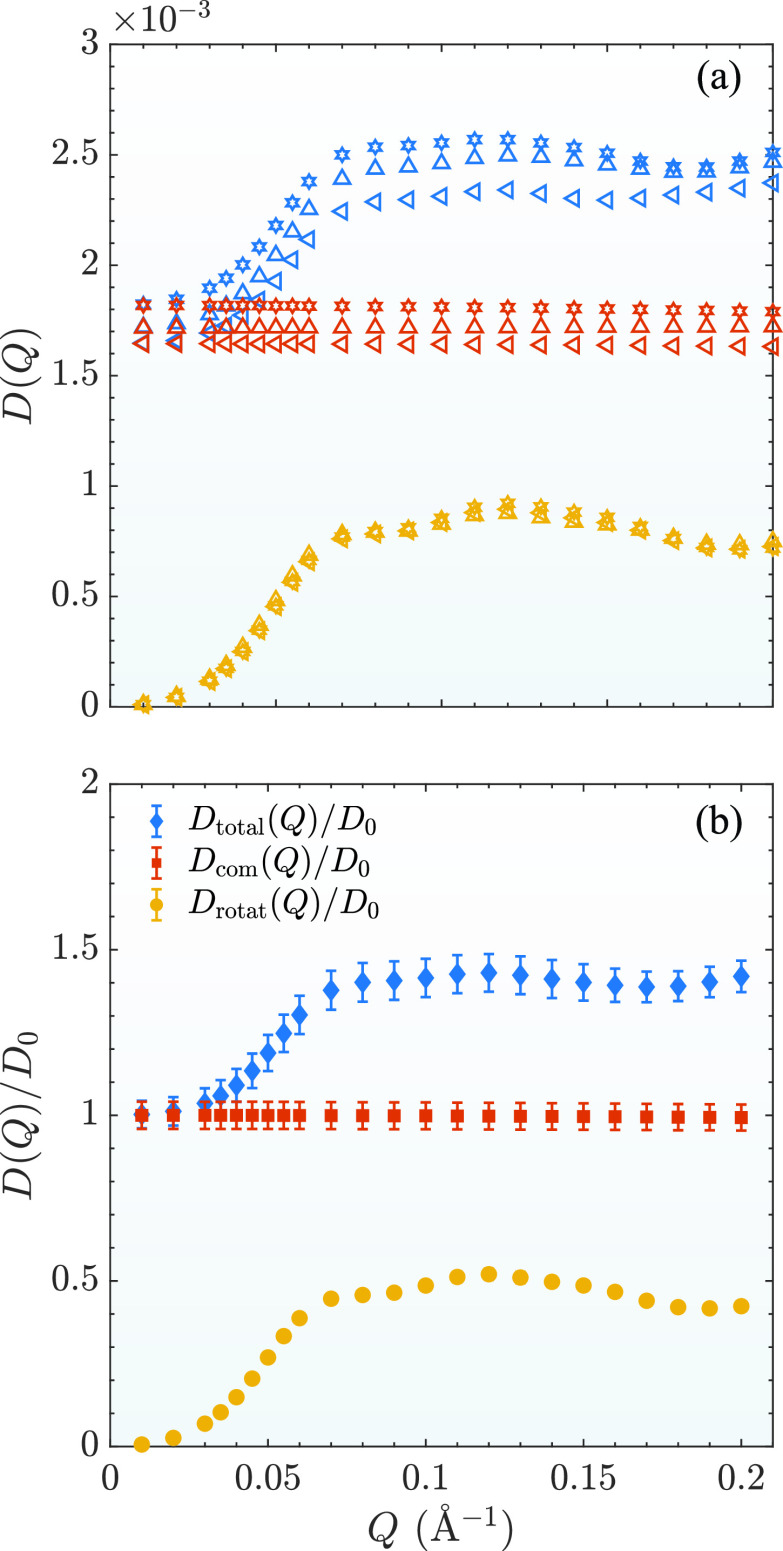
(a) Total (blue symbols), center-of-mass (red symbols), and rotational (yellow symbols) diffusion coefficient for three different trajectories differentiated by the upper and lower triangles and stars calculated by fitting the corresponding ISF to Eq. (15) within the time region showed in Fig. 4. (b) Corresponding averaged effective, center-of-mass, and rotational diffusion coefficients. The isotropic free diffusion coefficient at the infinite dilute condition, *D*_0_, which represents the diffusion coefficient at infinitesimal *Q*, is taken as $D_{\text{trans}}$ at *Q* = 0.01 Å^−1^.

The effective total ($D_{\text{total}}(Q)$), center-of-mass ($D_{\text{com}}(Q)$), and rotational ($D_{\text{rot}}(Q)$) diffusion coefficients as a function of *Q* are shown in Fig. 5(a) from different trajectories. $D_{\text{rot}}(Q)$ is almost identical for all three trajectories. However, even though $D_{\text{total}}(Q)$ and $D_{\text{com}}(Q)$ from different trajectories are similar to each other, the difference from different trajectories is about 10%. Note that unit used in Fig. 5(a) is not converted to the diffusion of a mAb in a real system. To compare the results with the experimental data, the results are normalized by the isotropic free diffusion coefficient at the infinite dilute condition by dividing $D_{\text{com}}(Q)$ at *Q* = 0.01 Å^−1^ that should be the same as *D*_0_. The results are shown in Fig. 5(b). The error bars are estimated based on the variation of the results from different trajectories of three independent simulations.

The approximation by Eq. (9) leads to the approximation of the short-time diffusion coefficients so that

$$D_{\text{total}}(Q)=D_{\text{com}}(Q)+D_{\text{rot}}(Q),$$ (17)

with

$$D_{\text{com}}(Q)={\left\langle D_{0}(\vec{Q})\right\rangle }_{\theta }=D_{0}$$ (18)

for a rigid particle at the dilute condition. However, it is useful to point out that the exact result for $D_{\text{total}}$ should be^17,25^

$$D_{\text{total}}(Q)=D_{\text{trans}}(Q)+D_{\text{rot}}(Q),$$ (19)

where

$$D_{\text{trans}}(Q)=\frac{\langle F(\vec{Q})F^{*}(\vec{Q})D_{0}(\vec{Q}))\rangle_{\theta }}{P(Q)}$$ (20)

if the rotational and center-of-mass motions are independent of each other. Both $D_{\text{com}}$(Q), and $D_{\text{trans}}$(Q) are related to the translational motion only. However, different from $D_{\text{com}}$(Q), $D_{\text{trans}}$(Q) couples the motions with the shape of the particle through Eq. (20).

**FIG. 6.**
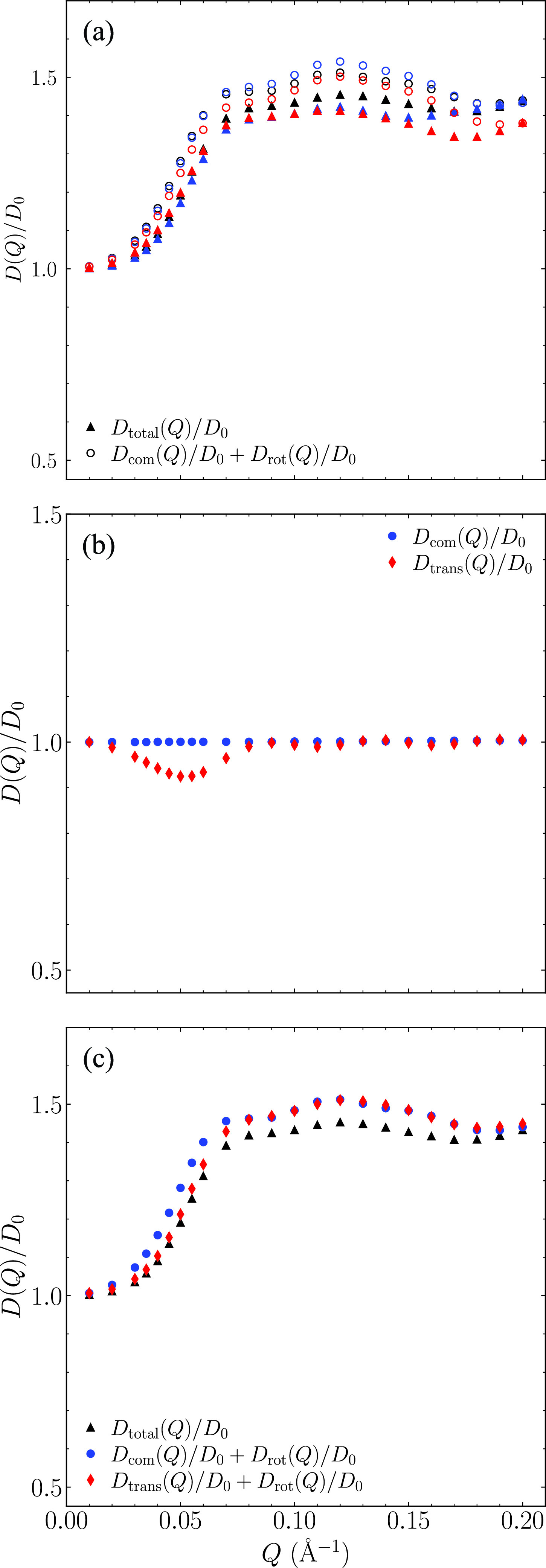
(a) Comparison between $D_{\text{total}}$ calculated directly from Eq. (5) and $D_{\text{com}}+D_{\text{rot}}$ for three independent trajectories. (b) Comparison between $D_{\text{com}}$(Q) and $D_{\text{trans}}$(Q). (c) Comparison between $D_{\text{total}}$ with those approximated using Eqs. (17) and (19).

The accuracy of Eq. (17) is first evaluated by comparing $D_{\text{total}}(Q)$ with $D_{\text{com}}(Q)+D_{\text{rot}}(Q)$ as shown in Fig. 6(a). Even though the difference is overall small, the results from all three trajectories show a consistent discrepancy. It is found that the simple addition of $D_{\text{com}}(Q)$ and $D_{\text{rot}}(Q)$ overestimates the value of $D_{\text{total}}(Q)$ with the largest difference up to about 10%.

To further understand what causes the difference, one trajectory of our simulations is further investigated. The calculated $D_{\text{com}}(Q)/D_{0}$ and $D_{\text{trans}}(Q)/D_{0}$ from this particular trajectory are shown in Fig. 6(b). (The results calculated from other trajectories are similar.) It is not surprising that $D_{\text{com}}(Q)/D_{0}$ is constant while $D_{\text{trans}}/D_{0}$ changes as a function of *Q*. Overall the difference between these two coefficients are very small. The largest difference is about 10% at around Q≈ 0.05 Å^−1^, which approximately correspond to the length scale of the diameter of the protein.

Figure 6(c) shows the results of $D_{\text{trans}}(Q)+D_{\text{rot}}(Q)$ so that we can evaluate the accuracy of Eq. (19). For *Q* < 0.07 Å^−1^, $D_{\text{trans}}(Q)+\;D_{\text{rot}}(Q)$ is almost identical with $D_{\text{total}}(Q)$. However, it becomes larger than $D_{\text{total}}(Q)$ at larger *Q* values. The largest difference is about 4%. Therefore, Eq. (19) is more accurate than Eq. (17) at the small and intermediate *Q* values. However, both overestimate the total diffusion coefficient at larger *Q* values (*Q* > 0.07 Å^−1^). This indicates that for larger *Q* values, the translational and rotational motions are not completely decoupled at the short time limit as described by either Eq. (17) or Eq. (19).

Note that Eqs. (19) and (20) are equivalent to that proposed in Ref. 17 if the rotational and translational motions are independent of each other. Since Eq. (19) has been widely used,^17,18^ we therefore need to be prudent when studying some small differences of mAb proteins using NSE with this approximation. For many flexible proteins, the deviation of measured diffusion coefficient from that of the rigid protein model has been attributed to the internal domain motions. Our results show that using Eq. (19) may potentially affect the accuracy of estimated internal domain motions. Equation (17) is shown to be less accurate than Eq. (19). Even though Eq. (17) is less frequently used to model the diffusion coefficient of flexible proteins, it is implicitly used in Eq. (9) that has been widely used for many studies.^20,23,26^ Because the better agreement between $D_{\text{total}}(Q)$ and $D_{\text{trans}}(Q)+D_{\text{rot}}(Q)$, it is reasonable to propose that Eq. (9) could be replaced with

$$\frac{I(Q,t)}{I(Q,0)}\approx I_{\text{trans}}(Q,t)I_{\text{intra}}(Q,t),$$ (21)

where $I_{\text{trans}}(Q,t)=e^{-D_{\text{trans}}(Q)Q^{2}t}$.

**FIG. 7.**
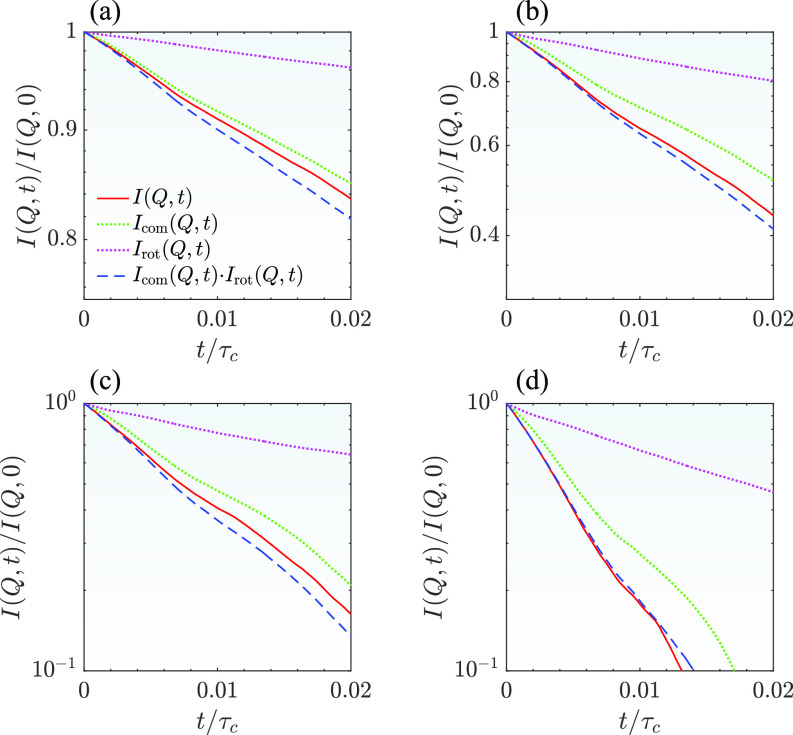
Comparison between total collective ISF and the product of the center-of-mass and rotational ISFs at different *Q* values. (a) *Q* = 0.05 Å^−1^, (b) *Q* = 0.1 Å^−1^, (c) *Q* = 0.15 Å^−1^, and (d) *Q* = 0.2 Å^−1^.

We further evaluate the accuracy of the intermediate scattering function using Eq. (9) at a finite correlation time as this approximation has been widely used.^20,26^ Figure 7 shows the results of $I(Q,t)/I(Q,0),\;I_{\text{com}}(Q,t)/I_{\text{com}}(Q,0)$, and $I_{\text{rot}}(Q,t)/I_{\text{rot}}(Q,0)$ together with the multiplication of $I_{\text{com}}(Q,t)/I_{\text{com}}(Q,0)$ and $I_{\text{rot}}(Q,t)/I_{\text{rot}}(Q,0)$ calculated from one trajectory. Even though the uncertainty may be large for the intermediate scattering functions at large correlation time as demonstrated in Fig. 3, all trajectories are still subject to the same physics. It is still possible to evaluate the coupling of the rotational and translational motions even with one trajectory. Overall, simply decoupling between the center-of-mass and rotational motions by multiplying the intermediate scattering functions together shows a reasonable agreement with I(Q,t)/I(Q,0) at all four *Q* values. However, there is still small difference between them. We have calculated the results from other trajectories (not shown here). The results all show similar results as shown in Fig. 7. Hence, similar to the comparison of the short-time diffusion coefficients, we need to be prudent when interpreting a subtle difference by comparing the results from the decoupling approximation using Eq. (9) with that from an experiment. Overall, not surprisingly, the center-of-mass motions contribute more to the relaxation of *I*(*Q*, *t*) as a function of time compared to that of the rotational motions. As the experimental error bars of a NSE experiment on a mAb system are usually larger than the difference demonstrated here in Fig. 7, the approximation by Eq. (9) seems to be reasonable.

## V. CONCLUSIONS

The dissipative particle dynamic simulation is conducted for a rigid-body model of the NISTmAb. The intermediate scattering functions and the effective short-time diffusion coefficients are calculated based on the trajectories of the simulations. It is found that in order to obtain reliable values of the intermediate scattering functions for our systems, the simulation time has to be about two orders of magnitude longer than the interested correlation time.

A commonly used approximation by decoupling the center-of-mass and rotational motions of a protein is evaluated for this model mAb protein. This decoupling approximation is observed to overestimate the total effective diffusion coefficient. Approximating the translation motion using $D_{\text{trans}}(Q)=\frac{{\left\langle F(\vec{Q})F^{*}(\vec{Q})D_{0}(\vec{Q})\right\rangle }_{\theta }}{P(Q)}$ instead of using $D_{\text{com}}$ shows much better agreement with the total diffusion coefficient using the Eq. (19). However, at relatively high *Q* values, both Eqs. (17) and (19) overestimate the total diffusion coefficient indicating that the translational and rotational motion are not completely decoupled. However, overall, Eq. (19) has a better agreement than that calculated using Eq. (17). Also, the difference due to the two different approximations is relatively small over the studied *Q* range with the largest difference to be about 10% at about the *Q* value corresponding to the diameter of the protein.

The estimated total intermediate scattering function is different from the intermediate scattering function calculated based on the decoupling approximation [Eq. (9)]. However, the overall agreement is still not so bad. As the error bars of many NSE experimental results are relatively large, the difference introduced by the decoupling approximation could be still considered acceptable.

Many studies of the internal motions of proteins using NSE need an accurate estimation of the NSE signals of a rigid body protein model. Our results demonstrate that the assumption of the decoupling between the center-of-mass and rotational motions could introduce appreciable errors to the data interpretation. It is also important to point out that the coupling between the center-of-mass and rotational motion is expected to depend on the overall shape of a protein. Thus, the quantitative difference due to the coupling approximation could be different for other proteins with different shapes. In addition, when the protein is very flexible, its internal motions could further potentially affect the coupling between the rotational and translational motions that may need future quantitative studies.

## Data Availability

The data that support the findings of this study are available from the corresponding author upon reasonable request.
